# Radon exposure risks among residents proximal to gold mine tailings in Gauteng Province, South Africa: a cross-sectional preliminary study protocol

**DOI:** 10.3389/fpubh.2024.1328955

**Published:** 2024-03-08

**Authors:** Khathutshelo Vincent Mphaga, Wells Utembe, Phoka Caiphus Rathebe

**Affiliations:** ^1^Department of Environmental Health, Faculty of Health Sciences, Doornfontein Campus, University of Johannesburg, Johannesburg, South Africa; ^2^National Health Laboratory Service, Toxicology and Biochemistry Department, National Institute for Occupational Health, Johannesburg, South Africa

**Keywords:** indoor radon exposure, lung cancer, COPD, Leukemia, gold-mine tailings, AlphaE radon monitor

## Abstract

Gold mine tailings, a legacy of the mining industry, harbors significant amount of radon gas, a classified human carcinogen. Radon exposure, especially near tailings, is a significant public health threat, potentially leading to increased risk of lung cancer, leukemia, and chronic obstructive pulmonary disease (COPD). These health problems are often associated with lower survival rates and significant financial burdens. This ongoing research aim to evaluating the relationship between indoor radon exposure and lung cancer, leukemia, and COPD risks among residents proximal to gold mine tailings in Gauteng Province, South Africa. This cross-sectional preliminary study focus on two distinct groups: Riverlea (exposed group, <2 km to Gold mine tailings) and Orlando East (unexposed group, >2 km to Gold mine tailings). Indoor radon levels is measured using AlphaE monitors, while health risks (lung cancer, leukemia, and COPD) linked to exposure are evaluated through interview-administered questionnaire and secondary data from Gauteng Health Department. Of the 476 residents randomly selected for this study, 300 have already participated, with balanced representation from both the exposed and unexposed groups. The study will compare indoor radon levels and health outcomes between the two groups. This study’s results could aid in creating targeted interventions and policies to mitigate indoor radon exposure risks and safeguard vulnerable communities from this significant public health hazard.

## Introduction

In South Africa (SA), particularly in Gauteng province, most human settlements are close to gold mine tailings (1, 2). The province has more than 250 mine tailings; most of these are abandoned, unfenced, unguarded, unmaintained, and not vegetated, making them a substantial source of air and water pollution (3–5). Gold mine tailings account for almost half of all the waste generated in SA (5). It has been estimated that about 1.6 million people in Gauteng Province (particularly in Johannesburg) reside adjacent to mine tailings, despite international and local mining legislations prohibiting the establishment of human settlements proximal (<500 m) mine tailings (6–8). The proximity of many homes closer to Gold mine tailings has raised many concerns about the rights to health and a healthy environment due to documented links between proximity to mine dumps and increased respiratory diseases like asthma, pneumonia, and chronic bronchitis (1, 2, 4, 9–14).

The majority of the exposed communities are of lower socio-economic status, marginalized ethnic groups living in government-funded houses, and informal settlements (1, 2, 8–10). Residents residing in proximity to Gold mine tailings experience elevated radiation exposure owing to the presence of significant quantities of uranium and its decay products, notably radon, within the tailings (15, 16). This exposure frequently surpasses the annual safety threshold of 1 Millisievert (mSv) established by the World Health Organization (WHO) and the National Nuclear Regulator (NNR) (15, 17, 18). Radiometric surveys have played a crucial role in assessing environmental safety and preventing unknowingly exposure to excessive background radiation (15, 16). Natural radioactivity permeates rocks, water, soil, and living organisms, with decaying elements like uranium, influencing radiation levels. In anomalous zones, such as proximal gold mine tailings with higher concentrations of these elements can lead to severe health consequences due to radiation exposure. Uranium, which decays into radon, is of particular concern as extended exposure to uranium has been associated with leukemia, hepatic and kidney diseases, liver damage, and internal organ dysfunction (13, 18).

Radon is a chemically inert radioactive gas formed naturally across the earth’s crust by the radioactive decay of uranium and radium (19, 20). Radon has a half-life of 3.8 days, allowing it to diffuse through the soil and into the air before decaying into other radioactive substances called Radon Daughter Progenies (RDPs) (21). Radon gas can be found in rocks, soil, building materials, water, air, and natural gas (19–21). Due to its extended half-life, radon readily attaches to airborne particles or geological materials, enabling its transport across the atmosphere for both short (less than 2 km) and long distances (up to 20 km), as reported in various studies (6–8, 22, 23). Radon gas enters a building through various routes, including cracks in floors, construction joints, walls, suspended floors, service pipes, and cavities in walls (6–8, 17–24). International organizations such as WHO, United States Environmental Protection Agency (EPA), and International Agency for Research on Cancer (IARC) has declared indoor radon a human carcinogen (25). Radon decay products inhaled from the air constitute the primary source of radiation exposure for the global population (26). Several large-scale epidemiological studies found a statistically significant linear relationship between lung cancer risk and cumulative radon exposure (24, 27, 28). According to WHO, radon is considered the second leading cause of lung cancer among smokers and the leading cause of lung cancer among non-smokers (11, 15, 19, 25–27). Furthermore, radon is responsible for 3–14% of lung cancer cases (28–34). Other studies have concluded that indoor radon exposure is responsible for 3–20% of all lung cancer deaths worldwide, depending on a country’s average radon level and smoking prevalence (20–28). It has been estimated that for every 100 Becquerel’s per cubic meter (Bq/m^3^) increase in long-term radon exposure is estimated to raise the relative risk of lung cancer by 16% (35, 36). The risk of dying from lung cancer have been found to be higher in residential areas situated less than 5 km from mines (29). The global burden of lung cancer mortality is gradually increasing (37). Hence, WHO rated lung cancer as the leading cause of death caused by cancer in 2020 (21). In SA, lung cancer was the most significant cause of cancer-related death, accounting for 7,730 deaths in 2020 (38). Radon studies indicate that radon increases lung cancer risk without a threshold and can be carcinogenic at any level (17, 21). The economic burden of lung cancer treatment in South Africa is substantial, with an average cost per patient of R620,000, resulting in an annual cost of R 2.2 billion (39). While the link between radon exposure and lung cancer is undeniable, its association with other cancers (skin, stomach, blood, pancreatic, and liver) lacks conclusive evidence (31). Radon exposure has also been tentatively linked to COPD, leukemia, dementia, asthma, bronchitis, and fibrosis, but further research is needed to solidify these connections (16, 31, 40–45), Notably, factors like smoking and dust exposure could potentially influence these observed health outcomes.

Proximity to gold mine tailings, a prime source of radon gas, puts residents at higher risk of elevated indoor radon levels compared to the general population (46–51). Indoor radon exposure is a serious concern because people spend most of their time indoors (i.e., 80%), and if radon concentration in such settings is at a dangerous level, it could result in adverse health effects (17, 22, 52). Despite claiming twice, the lives of drunk driving and exceeding homicides in United States, radon gas, the leading in-home hazard, remains a neglected public health threat (53, 54). To combat radon-induced illness, identifying populations with high indoor radon exposure and implementing preventative measures is crucial (17, 22). Limited research on the impact of residential radon exposure near gold mine dumps necessitates investigating its potential health risks in South African communities. While health implication of radon exposure in occupational settings (i.e., among miners) have received overwhelming attention, the health implication of residing proximal radioactive sites or mine fields (i.e., gold mine tailings) of remains largely unexamined (26, 46). This study aims to address this gap by exploring the local context and potential health consequences of radon exposure in these communities.

South Africa’s unique situation requires a separate investigation into radon exposure, despite the availability of global studies. While the warm climate might promote better ventilation and lower radon levels indoors, the cold winters and lack of heating in Highveld region (i.e., Gauteng) could lead to stagnant indoor air, potentially trapping radon (26, 46). Additionally, densely populated areas around Witwatersrand are surrounded by gold and uranium mine waste dumps with high radon concentrations. These contrasting factors necessitate a local study to understand the true impact of radon exposure in South Africa [6–8.27]. A critical gap in knowledge exists regarding the health risks associated with indoor radon exposure near gold mine dumps in South Africa. The potential link between radon and lung cancer, leukemia, and COPD remains unclear, particularly in Gauteng, a province that houses numerous Gold mine tailings. Unlike other countries (mostly developed), South Africa lacks government monitoring of indoor radon levels, leaving residents unaware of their exposure. Therefore, it is in the public’s interest to establish the health implication of everyday indoor radon exposure, particularly among people residing proximal radioactive sites (i.e., gold mine tailings) (15, 18, 26, 30, 50).

The imperceptibility of radon, characterized by its absence of color, odor, and taste, necessitates measurement as the sole means of determining exposure levels. While many countries prioritize residential radon monitoring, South Africa, especially Gauteng with its abundance of mine dumps, has largely neglected this issue (3–5). Furthermore, buildings construction practices near these mine dumps raise concerns, as building contractors and residents sometimes utilize radioactive tailing sand, a potential carrier of radon gas, for building their homes, despite current legislations (Regulation No 388 of 2006) prohibiting such practices (1, 2). This study aims to shed light on this understudied yet critical public health concern by investigating the association between indoor radon exposure and self-reported lung cancer, leukemia and COPD in Gauteng communities residing near mine tailings.

The objectives of this study are:

- To quantify radon concentration levels in residential houses proximal to gold mine tailings.
- To determine the association between indoor radon exposure and self-reported lung cancer risk.
- To determine factors associated with high indoor radon concentrations and self-reported lung cancer prevalence in residential houses proximal to gold mine tailings.
- To determine the association between indoor radon exposure and self-reported COPD and leukemia risks

## Methods and analysis

### Study design

This study investigate the potential link between indoor radon exposure and self-reported lung cancer, leukemia, and COPD in Gauteng, South Africa. Gauteng is the country’s most populous province with over 15.8 million residents (55). Utilizing a cross-sectional preliminary design, this ongoing study will compare indoor radon levels and self-reported health issues among residents living near and distal gold mine tailings in Riverlea (proximity) and Orlando East (distance), offering a preliminary assessment of the association.

### Exposed and unexposed population

Residents living within 0.1–2 km of the gold mine tailings in Riverlea, South Africa, are considered the exposed group in this study. Located south of the Mooifontein Gold mine tailings, Riverlea is a 3.4 km^2^ area within the City of Johannesburg (COJ), region B. Riverlea, a densely populated community of 16,226 residents in 4,208 dwellings, sits precariously next to three active and dormant gold mine dumps (2, 4, 49). This proximity, coupled with ongoing gold recovery efforts and arid conditions, has resulted in persistent dust pollution and hindered rehabilitation efforts, leaving the partially vegetated tailings as a potential health hazard (2, 4). Previous research has identified an increased risk of respiratory problems (i.e., cough and asthma) in Riverlea (8, 9). Therefore, considering the geographical location, environmental factors, and existing health concerns, Riverlea serves as a representative population potentially exposed to higher levels of indoor radon compared to the control group, making it an ideal choice for this study.

Orlando East, located 5 km from Riverlea, is considered an unexposed group due to the absence of gold mines or mining history that could be linked to radon exposure. The nearest gold mine tailings are over 2 km away. The community, which is part of the City of Johannesburg metropolitan area, region D, has a population of 68,210 and 22,416 households (56, 57). Demographic data further indicate that there are 4,135 erven designated for residential purposes within this community (57). Sharing similar demographics, socio-economic background, and residency in state dwellings, both Riverlea and Orlando East represent comparable communities historically facing disadvantages (9, 10, 58). This similarity allows for effective control when comparing indoor radon levels. Since radon concentrations typically elevate near gold mine tailings due to uranium presence, contrasting indoor radon levels between Riverlea (proximity to tailings) and Orlando East (distant from tailings) offers a reliable method to pinpoint potential radon sources and evaluate exposure risks.

### Sample size and sampling procedure

**Figure 1:**
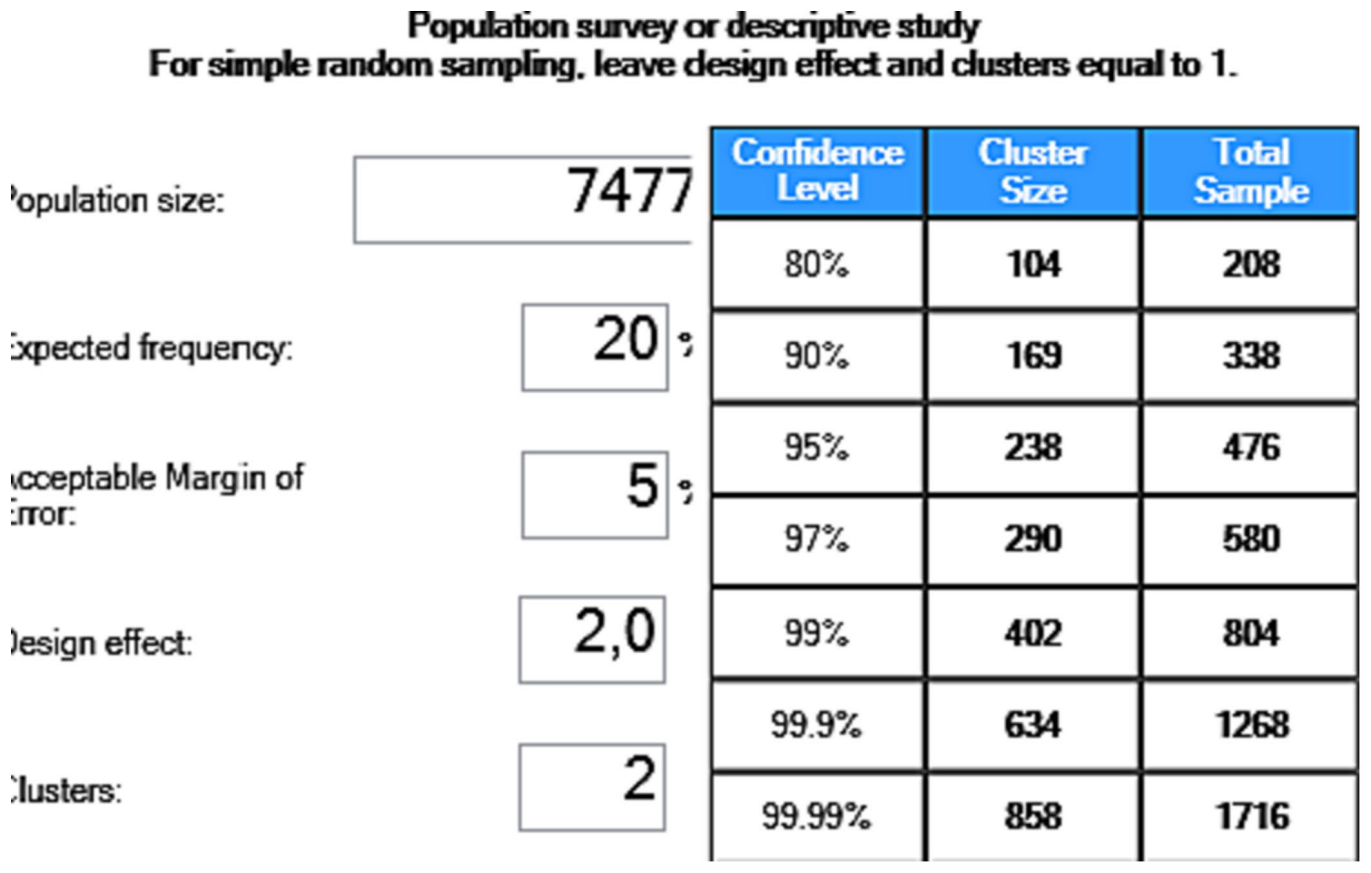
Sample size estimation using EPINFO 7.

Considering data from Johannesburg online maps indicating 7,477 residential erven across Riverlea and Orlando East, the study population was set at 7,477, assuming one potential participant per dwelling. This aligns with the focus on dwelling characteristics influencing indoor radon levels rather than individual occupants (17, 22). Given the unavailability of local cancer statistics (59, 60), the established international lung cancer incidence rate of 20% was used to calculate the required sample size (20, 28). Utilizing the “EpiInfo” software for two population groups with a 20% expected frequency, 95% confidence level, and 5% margin of error, a sample size of 476 residents (238 each from Riverlea and Orlando East) was determined. This calculation is visualized in Figure 1.

## Inclusion and exclusion criteria

The study aims to accurately represent the target population and minimize bias by using inclusion and exclusion criteria. Only one resident per dwelling, over 18 years old, residing in a ground-level home within the study area is eligible. This study requires voluntary participation and informed consent. Before deciding, potential participants is fully briefed on both the potential benefits and risks involved. Additionally, the study focuses on dwellings with specific characteristics known to influence indoor radon levels, such as direct contact with the ground. This ensures the collected data accurately reflects real-world conditions with the highest exposure potential. Minors (below 18 years old) are excluded to avoid complexities surrounding research with children. Additionally, individuals who decline consent are excluded to ensure data collection from genuinely interested participants likely to provide accurate and complete information. Furthermore, only ground-level dwellings with direct earth contact are included, as housing characteristics like wooden structures, upper floors, and non-residential buildings are known to have minimal influence on indoor radon levels. This focused approach prioritizes data reflecting real-world conditions with the highest exposure potential.

## Data collection

### Data collection team

The researchers, accompanied by two trained research assistants fluent in local languages (Afrikaans in Riverlea and isiZulu in Orlando East), are conducting data collection. Equipped with interviewer-administered questionnaires and AlphaE radon monitors, the aim is to gather information from 476 participants. Global Positioning System (GPS) technology is used to locate difficult-to-find houses.

### Recruitment procedure and data collection period

To recruit participants, the study is using a random sampling approach. Residential dwelling lists has been obtained from the COJ website, forming the sampling frames for both strata with equal representation (57). Utilizing Microsoft Excel, the researcher randomly selected participants from each community based on stand numbers, and street addresses. The resulting participant list, along with dwelling street addresses, is used to facilitate the organization of daily research routes for efficient data collection. Once a randomly selected house is located, the research team approaches the homeowner and explains the study’s purpose in detail. If consent is granted, the interviewer administers a questionnaire to gather information about the household’s demographics, lifestyle habits (including ventilation and smoking), occupational background, and reported lung cancer risks. An AlphaE monitor is then installed in the home to measure indoor radon exposure for 2 h. Data collection for this study is ongoing. To date, 300 residents from the two communities have participated. The remaining residents, living near and far from the gold mine tailings, will be included in the study from June to September 2024. In South Africa, June–September represents the driest months, with low precipitation, allowing dust to be blown from Gold mine tailings easily to the nearby residential houses (1, 2, 5). Buildings are often less ventilated during this period than during the summer; therefore, it is recommended that radon measurements be done during the winter season to acquire maximum radon concentrations (18, 22).

### Data collection instruments

#### Questionnaire

To gather comprehensive data on potential factors influencing radon exposure and associated health effects, this study utilizes a researcher-administered questionnaire (see Supplementary material). Developed with consideration for participants’ literacy and language needs, the questionnaire covers socio-demographics, dwelling characteristics, occupational history, smoking habits, and health issues relevant to indoor radon exposure (22). This resource-efficient approach minimizes the need for follow-up while capturing rich information that may explain variations in observed radon levels and self-reported lung cancer risks. Data from the questionnaire will not only identify dwelling features and other factors potentially influencing radon concentration variability but also contribute to analyzing the relationship between indoor radon exposure (independent variable) and self-reported lung cancer (dependent variable). Recognizing the value of validated questionnaires in numerous countries, this study adapts a previously validated instrument used in a Canadian indoor radon study (22). This tailored approach ensures relevance and accuracy for gathering data within the study context.

#### AlphaE radon monitor

**Figure 2:**
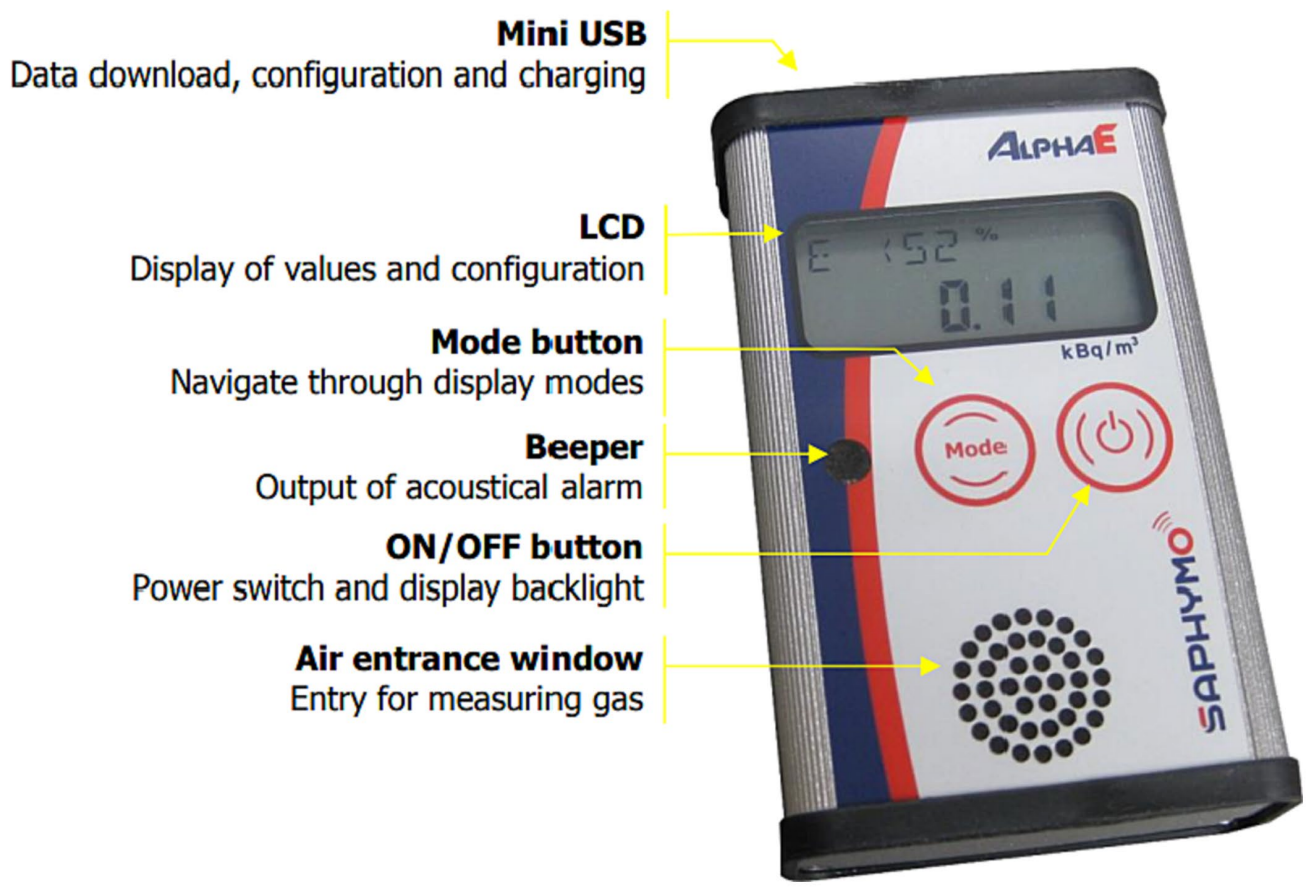
AlphaE radon monitor functional and control elements (50).

Measuring radon concentration is crucial for understanding health risks and designing mitigation strategies. This study uses the AlphaE monitor (shown in Figure 2), a portable device capable of detecting radon as low as WHO recommended guidelines limits of 100 Bq/m^3^ (17, 22). AlphaE radon monitors used in this study has been independently evaluated by multiple organizations and found to be highly effective in detecting and measuring radon (61–63). AlphaE radon monitors were calibrated by exposing them to a known radon concentration for 48 h, with a reference unit (AlphaGUARD). The results showed AlphaE could accurately measure radon levels across a wide range, with a precision of ± 10% at the 95% confidence level. The AlphaE works by detecting alpha radiation from radon decay and offers features like real-time monitoring, low-level sensitivity, and data storage. While its response to concentration changes is slower than some monitors due to its sensitivity to all alpha particles, its fast component reaches 50% equilibrium in 20 min and 90% in 60 min, making it suitable for short-term, integrated measurements (61–63). The AlphaE’s compact size, real-time monitoring capabilities, and minimal radiation effects render it ideal for research purposes, allowing for efficient data collection in various locations within homes and workplaces.

During the winter months of June and July 2023, indoor radon levels were measured in the participants’ homes using four AlphaE radon monitors belonging to the University of Johannesburg. As of now, 300 indoor radon measurements have been completed, with the remaining dwellings scheduled for assessment between June and September 2024 to achieve the desired sample size. The AlphaE monitors is set to test indoor radon concentrations every 10 min for 2 h. To accurately capture typical occupant exposure, AlphaE monitors are placed in stable locations within frequently occupied rooms, maintaining distance from walls, doors, windows, and potential interference sources like heat or airflow. Measurements are recorded in Bq/m^3^ and transferred digitally to a computer for analysis using DataVIEW communication software.

#### Secondary data

Secondary data on health problems (lung cancer, leukemia, and COPD) associated with indoor radon exposure has been requested from the Gauteng Health Department. This data will be used to assess if there is a linked between high indoor radon exposure proximal to gold mine tailings and the associated health outcomes. Primary data on health problems collected through a questionnaire will also be used to evaluate the relationship between radon exposure and associated health problems. A letter has been sent to the Gauteng Health Department requesting secondary data for the two communities. The requested information will include the residential addresses of the cases, their ages, occupational histories, and smoking histories. Only lung cancer cases reported in the past 20 years will be needed.

## Pilot study

A pilot study was conducted on June 20, 2023, to test the logistical and practical aspects. The pilot study used a 47-question interviewer-administered questionnaire to collect data from 16 randomly selected residents (eight from Riverlea and eight from Orlando East). Most participants found the questionnaire to be easy to understand and relevant. However, some questions were revised based on participant feedback. Specifically, the question on the main materials used to build a house was revised to focus solely on the materials used to construct the roof. Furthermore, the pilot study results suggested that the main survey should avoid asking questions about combined household income and soil type, as these questions were found to be difficult or impossible for participants to answer. Pilot study data reliability was confirmed by Cronbach’s alpha of 0.7 in SPSS, exceeding the recommended threshold of 0.7 for good internal consistency.

## Study variables

Independent/explanatory variable: indoor radon concentration is our independent variable and is measured using AlphaE radon monitors.
Dependent/outcome variable: the dependent variables are lung cancer, leukemia, and COPD. The dependent variables are measured using a questionnaire and secondary data from the Provincial Department of Health.
Confounding variables: potential confounding variables in the study includes tobacco smoking, and occupational history. Confounding variables are measured using a research questionnaire.

## Data analysis

The questionnaire and radon concentration data are captured manually into Statistical Package for Social Sciences (SPSS) version 29 for analysis. The data will be analyzed in line with the study objectives with the support of the biostatistician. Data analysis will be completed September 2024.

### What is the level of the radon concentration level in residential houses proximal to gold mine tailings?

Independent variable: proximity to gold mine tailings.
Confounding variables: residential dwelling characteristics, tobacco smoking, and water source.
Outcome variable: indoor radon concentration proximal to gold mine tailings, indoor radon concentration levels distal gold mine tailings.
Statistical analysis: descriptive statistics will be used to profile both groups by analyzing all variables. Categorical data will be summarized with percentages, frequencies, and cross-tabulations. Continuous data will be described using mean (standard deviation) for normally distributed data and median (interquartile range) for non-normally distributed data. Shapiro–Wilk and Kolmogorov–Smirnov tests will be used to assess the normality of radon concentration levels. If the data are not normally distributed, the researchers will consider collapsing the continuous variable into a categorical variable (i.e., the proportion of indoor radon concentration will be divided into six pre-defined categories; 0–100, 101–149, 150–199, 200–249, 250–299, and ≥300 Bq/m^3^). If this transformation does not achieve normality, the researchers will use non-parametric tests. The Kruskal-Wallis test will be used to compare indoor radon concentration against proximity to gold mine tailings. A chi-square test of independence will be used to test if radon concentration is the same between residential houses proximal to gold mine tailings (<2 km) and residential houses far away (>2 km) from goldmine tailings.

### What is the risk of self-reported lung cancer associated with indoor radon exposure?

Independent variable: indoor radon concentration; proximity to gold mine tailings.
Confounding variables: socio-demographic, smoking habits, residential dwelling characteristics, and occupational characteristics.
Outcome variable: self-reported lung cancer risks proximal to gold mine tailings, self-lung cancer risks far away from gold mine tailings.
Statistical analysis: Chi-square test: the chi-square test will be used to assess the relationship between lung cancer risk and the independent variables, as well as the interaction between the two. Logistic regression analysis (LRA): odds ratios (ORs), adjusted odds ratios (AORs), and 95% confidence intervals (CIs) will be calculated using bivariate (univariate) and multivariate LRA to estimate the likelihood of having lung cancer among the study population. Independent variables with *p* < 0.20 in the bivariate LRA will be included in the multiple LRA. A *p* value <0.05 will be considered statistically significant in the multiple LRA. Effect modification: effect modification between indoor radon exposure and self-reported lung cancer risk, as well as other confounding factors (e.g., sex, age, tobacco smoking, occupational history, and type of fuel used at home), will be investigated by including a multiplicative term in the model.

### What factors contribute to high indoor radon concentration and lung cancer risks in residential houses proximal to gold mine tailings?

Independent variable: proximity to gold mine tailings, socio-demographic characteristics, residential house characteristics, tobacco smoking, and occupational exposure characteristics.
Outcome variable: indoor radon concentration and self-reported lung cancer risks.
Statistical analysis: To investigate the relationship between radon concentration and a range of potential predictors, bivariate and multivariate logistic regression analysis (LRA) will be used to calculate odds ratios (ORs), adjusted odds ratios (AORs), and 95% confidence intervals (CIs). Univariate and multivariate LRA will be used to calculate ORs, AORs, and 95% CIs to test for factors associated with self-reported lung cancer (using explanatory variables). A *p* value < 0.05 will be considered statistically significant. The effect modification between proximity to gold mine tailings and other factors (household income, tobacco smoking, and residential house characteristics), which may influence radon concentration, will be investigated. Using multiple logistic regression, the effect modification between proximity to gold mine tailings and other factors (sociodemographic characteristics, tobacco smoking, occupational characteristics, and residential house characteristics), which may influence self-reported lung cancer, will be investigated.

### What is the risk of other health problems such as COPD and Leukaemia are associated with indoor radon exposure?

Independent variable: indoor radon concentration; proximity to gold mine tailings.
Confounding variables: socio-demographic, tobacco smoking, and occupational history.
Outcome variable: self-reported COPD and leukemia proximal to gold mine tailings, Self-COPD, and leukemia prevalence far away from gold mine tailings.
Statistical analysis: self-reported COPD and leukemia incidences will be stratified by proximity to gold mine tailings. A chi-square test will be applied to determine the relationship (and interaction) between COPD and leukemia prevalence and independent variables. Odds ratios (ORs), adjusted odds ratios (AORs), and 95% confidence intervals (CIs) are calculated with univariate and multivariate logistic regression analysis (LRA) to estimate the likelihood of having COPD and leukemia between the study groups. Independent variables with *p* < 0.20 obtained in the bivariate (univariate) LRA are included in the multivariate LRA. In the multivariate LRA, a *p* value < 0.05 is considered statistically significant. Effect modification between indoor radon exposure and self-reported COPD and leukemia and other confounding factors such as sex, age, tobacco smoking, occupational history, and type of fuel used at home is investigated by including multiplicative terms in the model.

## Using indoor radon measurements results

### Dose estimation

The United Nations Scientific Committee on the Effects of Atomic Radiation (UNSCEAR) model will be used to determine the annual effective dose associated with indoor radon exposure. The annual effective dose is defined as the total dose of radiation that a person receives in a year, weighted for the different sensitivities of different organs and tissues to radiation (64). In this study, the annual effective dose due to inhalation of radon will be estimated using the measured indoor radon concentration. The effective dose represents the overall dose received by people in their homes. The calculation will be performed using the following equation (1), suggested by the UNSCEAR:


(1) $$ERn\;\left(\mathrm{mSv}.\;y^{-1}\right)=\mathrm{DCFRn}\;FRn\;ARn\;TRn\times 10^{-6}$$

where: *ERn* is the annual effective dose of inhaled radon;

*DCFRn* is the conversion factor of radon through inhalation (which is assumed to be 9 mSv/Bqhm^−3^);

*FRn* is the indoor equilibrium factor between radon and its daughters (which is assumed to be 0.4);

*ARn* is the activity concentration of radon in Bqm^−3^; and

Texp is the exposure time to the measured concentration (assumed to be 7,000 h in a year).

### Occupancy factor

The occupancy factor for the study area will be calculated using the following equation (2):


(2) $$\mathrm{Occupancy}\;\mathrm{factor}\left(\%\right)=\frac{\mathrm{mean}\;\mathrm{daily}\;\mathrm{occupancy}\left(h\right)\;X\;100\;\left(\%\right)}{24h}$$

### Development of indoor radon exposure map

Radon maps will be created using data obtained from participants, COJ Online Maps, and radon detectors. GIS software application will be used to create a radon map using the analyzed indoor radon exposure data and geocoded street addresses. The radon map will provide a visual depiction of radon levels across the study region, enabling the identification of areas with potential elevated radon exposure risks. Radon map will be developed immediately following data analysis in October 2024.

## Ethical consideration

The study is conducted in accordance with the University of Johannesburg ethical principles and guidelines. Ethical approvals were obtained from the University of Johannesburg Faculty of Health Sciences Research Ethics Committee (REC) (Clearance Number REC-1889-2023) and the Higher Degree Committee (HDC-01-115-2022).

### Informed consent

Permission has been obtained from gatekeepers, such as community leaders. Participants are provided with an information letter outlining the purpose, risks, benefits, confidentiality, and rights before signing an informed consent form. Participants are permitted to withdraw their consent prior to data submission, but due to the anonymous nature of the research, withdrawal of consent is not possible beyond this data submission.

### Privacy and confidentiality

The study ensures participants’ privacy by keeping their personal information private and confidential, using the data for statistical purposes only, and adhering to the Protection of Personal Information Act (POPIA). The researcher uses robust confidentiality measures, such as password-protected files, encryption, and locked storage, to protect participant identities. Personal identifying information is not collected, and participants are assigned unique codes. The study collects minimal demographic data and reports aggregate findings, making it inclusive of all relevant groups. The researcher’s commitment to confidentiality is reflected in the study’s ethical practices.

### Risks and benefits

The study is conducted ethically, without harm to participants, and is open to all groups. The findings will guide radon policies, allocate resources for remedial actions, and aid in developing future action plans. Participants benefit from the study by being informed about their home’s radon concentration and providing guidance on lowering it if necessary. The results may also be used to warn the public about potential health risks posed by radon.

## Discussion

The escalating public health concern surrounding indoor radon exposure necessitates immediate action (11, 13, 22, 23), particularly in South Africa where information on its magnitude and distribution is scarce (19, 26, 28, 30, 46). This lack of knowledge hinders the development of an effective radon action plan and leaves residents vulnerable to its detrimental health effects, including lung cancer, leukemia, and COPD (16–19, 22, 40). South Africa lacks published research assessing the health implications of indoor radon exposure (19, 39, 52, 53, 64–67). This critical knowledge gap necessitates urgent investigation to understand radon’s potential impact on public health, particularly given the prevalence of respiratory issues among residents residing near mine tailings (9, 10). Notably, radon exposure assessments in these areas remain largely neglected, despite their proximity to potential radon hotspots (19, 39, 64–67). Furthermore, South Africa lacks a national indoor radon map, unlike most countries, due to the absence of a comprehensive national survey as mandated by the IAEA (2, 18). This hinders targeted mitigation efforts and leaves the entire population susceptible to the unknown health risks posed by radon exposure (17, 22). The urgency to address South Africa’s radon knowledge gap is undeniable. This research intend to fill this critical void by investigating the magnitude and distribution of indoor radon exposure, particularly near mine tailings, and assessing its association with lung cancer, leukemia, and COPD. The findings of this study will provide invaluable scientific evidence to inform policy development and mitigation strategies, ultimately protecting public health from the detrimental effects of radon exposure.

While previous research on indoor radon and health relied heavily on lung cancer data and employed cohort, case–control, and correlation study designs (17, 22), this study takes a new direction. Due to the absence of reliable data on specific health outcomes of interest (59, 60), particularly near gold mine tailings, a robust case–control study is infeasible. Therefore, we opt for a cross-sectional approach. This, while not as rigorous as the aforementioned methodologies, allows for a snapshot comparison of exposure levels and health effects in the target population. This preliminary examination can offer valuable insights into the potential association between indoor radon and lung cancer, leukemia, and COPD, laying the groundwork for future studies using alternative designs to further validate or refine these initial findings. Participants will be recruited from residential houses proximal to gold mine tailings (exposed group) and an area with no history of mining (reference group). The distance for the exposed group has been set at less than 2 km, based on previous studies suggesting elevated radon concentrations and health risks in such proximity (19, 64–70).

While smoking plays a significant role in numerous health issues, including those under investigation like lung cancer, leukemia, and COPD, its impact should not overshadow the growing concern about indoor radon exposure (71). Radon is a significant lung cancer risk factor, ranking second among smokers and leading among never-smokers (70–74). This highlights the need to acknowledge the independent contribution of radon exposure to these health problems, alongside the established risks of smoking. The synergistic effect between radon exposure and smoking (active smoker, former smoker, and second-hand smoke) toward lung cancer is undeniable (17, 22, 30, 56, 75). Tobacco consumption, smoking intensity, duration, and time since quitting will be considered when calculating lung cancer, COPD, and leukemia risk. In addition, occupational and residential exposure to other carcinogens, such as asbestos, will be included in the analysis. Matching by geographical area and other socio-demographic characteristics, confounding will be controlled by considering common or possible risk factors for lung cancer, COPD, and leukemia. Information on the potential risk factors for all health problems under investigation will be obtained through a questionnaire.

Long-term radon tests (3–12 months) are preferred for estimating annual mean indoor concentrations due to their ability to account for the significant fluctuations that occur hourly, daily, weekly, and annually. This ensures a more accurate representation of residents’ true exposure levels (17, 22, 75, 76). Long–term radon tests may be time-consuming and require considerable human and financial resources. Long-term radon measurements, while ideal for individual assessments, hinder population-wide estimates due to limited sample size and potential volunteer bias (77). Practical challenges also arise: low participation rates and logistical constraints like lengthy mail-in kit analysis delays and lack of immediate feedback. These limitations impede representative data collection and risk skewing results in long-term studies (77–80). Hence, they are often utilized when short-term radon tests have shown elevated indoor radon concentrations (81, 82). Short-term radon tests, when conducted under closed-building conditions (in the winter season, when homes are closed up and radon is likely high), may provide valuable information during time-sensitive situations such as home sales, rapid initial screenings, and testing the effectiveness of the mitigation measures (77–81). Furthermore, they may give a first indication of the mean long-term radon concentrations in homes. Short-term radon tests are useful for screening purposes and may provide the first indication of radon problems. Due to budget constraints and time limitations, short-term radon tests will be conducted under closed-building conditions during the winter season. Studies of annual variation of radon concentration within the home environment suggest that elevated indoor radon concentrations are often observed during the winter season when windows and doors are kept closed (reduced ventilation) for a longer duration (17, 22, 77, 81–83). This approach will provide a first indication of potential radon problems and allows for faster results, enabling follow-up testing (using either short-term or long-term tests) and mitigation measures if necessary.

In terms of radon measuring device, this study replaces traditional track detectors (i.e., CR-39) (17, 22) with the more advanced AlphaE radon monitors due to their numerous advantages. Compared to CR-39’s delayed results and limited sensitivity, AlphaE shines with its real-time readings, providing crucial data within minutes. Its enhanced sensitivity tackles lower radon levels (20 Bq/m^3^, compared to CR-39’s 100 Bq/m^3^ detection limit), enabling early detection in areas of concern (61–63). Furthermore, AlphaE transitions from one-time measurements to continuous monitoring, revealing valuable insights into radon fluctuations and their environmental influences, ultimately optimizing mitigation strategies (81, 82, 84). The potential for smart home integration adds another layer of advantage, allowing for automated adjustments of ventilation or mitigation systems based on real-time readings. Beyond speed and sensitivity, AlphaE boasts shorter measurement periods (1 h–3 days) for accurate results compared to CR-39’s 3-month requirement, minimizing waiting time and uncertainty (61–63, 84). Additionally, it eliminates the need for chemical etching, simplifying the process and reducing hazards. Data storage and retrieval are also streamlined with digital storage, offering effortless access and analysis compared to manual track counting on CR-39 detectors (84). Despite the higher cost of AlphaE compared traditional monitors (CR-39), it is faster, more sensitive, and continuous monitoring capabilities, solidifying its position as a valuable tool for early detection, mitigation assessment, and smart home integration in radon management.

Self-reported questionnaires is used to collect data on human health problems associated with indoor radon exposure. This method is chosen due to its cost-effectiveness and ease of administration. Overall, this research design and methodology balance scientific rigor with practical considerations, aiming to provide valuable insights into the potential health risks associated with indoor radon exposure in communities residing near gold mine tailings. This research will pave the way for further investigations into the long-term health effects of radon exposure in these communities. Quantifying indoor radon levels, both near and far from gold mine tailings, can inform the development of environmental radiation exposure declaration. Such declaration may empower residents and authorities with informed location choices. Furthermore, the findings of this study may also be used to advocate for targeted interventions such as promoting radon mitigation measures in high-risk zones. By establishing the link between indoor radon exposure and various human health problems may enable identification of population group who are likely to develop various health problems, depending on where they stay. By identifying factors linked to high indoor radon exposure and associated health problem may contributes to the development of regulatory frameworks by guiding effective public health mitigation policies and resource allocation (56, 75).

### Strength and limitations

The use of a higher randomly selected sample of 476 dwellings in the current study will enhance accuracy as previous studies suggest that with only 100 randomly chosen samples, the percentage of dwellings with radon concentrations above the reference level can be determined to an accuracy of within about 25% (22, 52, 75). For a sample size of 50, this percentage can be determined only to an accuracy of within just over than 30% (22). Questionnaires have been widely used in many countries to gather useful information on the characteristics of dwellings, risk factors (i.e., tobacco smoking habits), and radon-induced health problems. Research questions were developed with the assistance of a statistician as recommended by the International Atomic Energy Agency (22, 75, 85). This will be the first local study to produce statistical data on indoor radon concentration and health problems related to it. As a result, data obtained from this study will provide information about indoor radon concentration, which will make people aware of potential radiological risks to their health. Also the data obtained may be used by the authorities and other stakeholders to set relevant regulations and conduct similar studies in other places with elevated uranium levels. Furthermore, it will assist experts and all affected parties to develop corrective measures to reduce radon exposure and associated risks. Since indoor radon concentrations will be measured under closed-building conditions, results may overestimate or overestimate annual mean radon concentration. It is important to remember that the results of radon measurements indoors made in one area of a country give no indication of the radon concentrations likely to be encountered in other regions. Since this study will take place primarily during winter, it will be impossible to account for seasonal radon variation (77–83). Despite the importance or benefits associated with the self-reported approach, some general limitations related to it do exist. These limitations include depending on participants’ memory to give the correct answer. In addition, they may avoid disclosing behaviors or illnesses that are stigmatized out of fear that such information may be disclosed (86). In order to obtain honest and open responses under such circumstances, participants will be assured that confidentiality and privacy with be kept throughout.

## Conclusion

While residents near gold mine tailings in Gauteng face a potential health risk from elevated indoor radon levels, the full extent and impact of this exposure remains unclear. This ongoing research is actively addressing this gap by quantifying radon concentrations in these communities. By investigating the association between radon exposure and self-reported lung cancer, COPD, and leukemia, the study aims to generate valuable data to inform future public health interventions and policy development. This information will not only empower residents with awareness of potential risks but also contribute to the creation of regional radon exposure maps, filling a crucial void in South Africa’s environmental health data. Ultimately, this research holds significant potential to improve public health outcomes in Gauteng by protecting vulnerable communities from the detrimental effects of radon exposure through targeted mitigation strategies and awareness campaigns.

## Data availability statement

The raw data supporting the conclusions of this article will be made available by the authors, without undue reservation.

## Ethics statement

The studies involving humans were approved by University of Johannesburg Faculty of Health Sciences Research Ethics Committee (REC) (Clearance Number REC-1889-2023) and the Higher Degree Committee (HDC-01-115-2022). The studies were conducted in accordance with the local legislation and institutional requirements. The participants provided their written informed consent to participate in this study.

## Author contributions

KM: Conceptualization, Data curation, Formal analysis, Funding acquisition, Investigation, Methodology, Project administration, Software, Writing – original draft, Writing – review & editing. WU: Conceptualization, Methodology, Supervision, Writing – review & editing. PR: Conceptualization, Methodology, Resources, Supervision, Writing – review & editing.
