# Supplementary material for: Radon exposure risks among residents proximal to gold mine tailings in Gauteng Province, South Africa: a cross-sectional preliminary study protocol
Source: Front Public Health. 2024 Mar 8;12:1328955. doi: 10.3389/fpubh.2024.1328955 (PMC10957527; doi:10.3389/fpubh.2024.1328955)
Supplement: Supplementary file 1 [file Data_Sheet_1.docx]

**Questionnaire**

| **The outcome of the questionnaire (*To be completed by the researcher*)** | |
| --- | --- |
| Questionnaire Number | ­­­­­___ of 500 |
| Completed |  |
| Not completed |  |
| The participant disagreed. |  |

House Number_______Location/Area________________________________________

This study aims to evaluate the association between indoor radon exposure and self-reported lung cancer amongst residential houses proximal to gold mine tailings in Gauteng. Radon is a radioactive gas that occurs naturally in soil, rocks, and building materials. Radon gas has been associated with lung cancer. Your household was randomly selected for this important study.

| 1. **Socio-demographic characteristics** | |
| --- | --- |
| 1. Dwelling location (by distance from the gold mine tailings) | 1. ☐ Riverlea; Near-field (0.1 – 2 Km) 2. ☐ Orlando East ( >2 Km from gold mine tailings). |
| 1. Gender: What is your gender? | 1. Male 2. ☐ Female 3. ☐ Not willing to Identify |
| 1. Age: What is your age? | 1. 39 Years or younger 2. ☐ 40 - 49 years 3. 50 - 59 years 4. 60 – 69 years 5. ☐ 70 - 79 years 6. ☐ 80 and above |
| 1. Marital status: What is your present marital status? | 1. Single, never married 2. ☐ Married 3. Divorced 4. ☐ Separated 5. ☐ Windowed |
| 1. Race: How do you describe yourself in terms of the population groups? | 1. Black African 2. ☐ Colored 3. Indian/ Asian 4. ☐ White 5. Other |
| 1. Educational level: What is your highest level of education? | 1. ☐ Less than high school 2. ☐ Completed high school 3. ☐ Completed tertiary education |
| 1. Employment status: Are you currently employed? | 1. Yes 2. ☐ No |
| 1. **Home Environment** | |
| 1. When did you start staying in this house? | 1. Less than 10 years ago 2. 11 – 20 years ago 3. 21 – 30 years ago 4. 31 years and more |
| 1. About how old is this building? | 1. 1 – 5 Years Old 2. ☐ 6 – 10 Years Old 3. 11 – 20 Years Old 4. 21 - 40 Years Old 5. Over 40 Year Old |
| 1. Which of the following best describes the main dwelling that this household occupies? | 1. Shed\Wendy House 2. Shack 3. Hut 4. Concrete\ brick house 5. Caravan 6. Other (specify below) ____________________ |
| 1. What is the main materials used for the construction of the roof of the dwelling? | 1. Corrugated iron/zinc 2. Asbestos roof 3. Tile 4. Other (specify below)   _____________________ |
| 1. What is the type of foundation of your home? | 1. Concrete slab 2. Stones and Mortar 3. Wood 4. Earth dirt 5. Other Specify____________ |
| 1. What of the following best describes the floor type of this dwelling that this house? | 1. Wood 2. PVC flooring 3. Tiles 4. Mud 5. Concrete 6. Carpet 7. Stone (marble, granite, slate, sandstone, limestone) 8. Other (Specify)_________ |
| 1. How many rooms are in this dwelling? | 1. 1 Room 2. 2 to 3 Rooms 3. 4 Rooms and above |
| 1. Does your house have any openings (or cracks) on the foundation or floor? | 1. Yes 2. ☐ No |
| 1. How many people live in this house | 1. One 2. 2 - 3 3. More than four |
| 1. How is water supplied to your home? | 1. Municipal distribution system 2. Private well water 3. Other (Specify)____________ |
| 1. What type of fuel or energy do you use? | 1. Electricity 2. ☐ Solar 3. Wood 4. Coal 5. Other (Specify)_____________ |
| 1. How often do you ventilate (such as opening windows) your home? | 1. Daily 2. ☐ More than once a week 3. Less than once a week 4. Only during certain activities 5. Do not ventilate at all |
| 1. How many hours do you spend inside the dwelling/ building per day? | 1. Less than 5 hours 2. ☐ 6 – 10 hours 3. 11 – 15 hours 4. 16 – 20 hours 5. 21 – 24 hours |
| 1. Has your home been tested for radon in the past? | 1. Yes 2. ☐ No |
| 1. **Occupational Environment** | |
| 1. Have you occupationally been exposed to | 1. ☐ Asbestos 2. ☐ Spray painting 3. ☐ Chromium planting 4. ☐ Melting of Asphalt, copper, nickel or aluminium 5. ☐ Coal 6. ☐ Dust 7. Diesel fumes 8. Coal smoke 9. None of the above |
| 1. If yes, how many years in total were you exposed? | 1. < 10 years 2. 11 – 21 years 3. 22- 32 years 4. 33 years and above |
| 1. Have you worked in underground mines? | 1. Yes 2. ☐ No |
| 1. If yes, how many years did you work in underground mines? | 1. ☐ < 10 years 2. ☐ 11 – 21 years 3. ☐ 22- 32 years 4. ☐ 33 years and above 5. None of the above |
| 1. **Smoking history** | |
| 1. Tobacco smoking | 1. Never Smoker 2. ☐ Ex-Smoker 3. Current smoker |
| 1. Does anyone in your household smoke? | 1. Yes 2. ☐ No |
| 1. How many people smoke in this home/ house? | 1. One 2. More than 2 3. None |
| 1. Do you or does any member of this house smoke inside the house? | 1. Yes 2. ☐ No |
| 1. How many cigarette did/do you smoke per day? | 1. None 2. < 10 Cigarettes/ day 3. ☐ 11- 21 Cigarettes/ day 4. 22 and above cigarettes/ day |
| 1. Total number of years smoking | 1. None 2. ☐ < 10 years 3. ☐ 11 – 21 years 4. ☐ 22- 32 years 5. ☐ 33 years and above |
| 1. **Health** | |
| 1. How difficult or easy is it to obtain the healthcare services? | 1. ☐ Very difficult 2. ☐ Difficult 3. ☐ Moderate 4. ☐ Easy 5. ☐ Very easy |
| 1. Have you ever been diagnosed with? | 1. ☐ Tuberculosis 2. ☐ Chronic bronchitis 3. ☐ None of the above |
| 1. Have you ever been diagnosed with any of the following conditions?   (Choose all health problems that are applicable) | 1. ☐ Asthma 2. ☐ Chronic obstructive respiratory diseases (COPD) 3. ☐ Leukaemia 4. ☐ None of the above |
| 1. Have anyone (i.e. children, parents, grandparents, brothers, sisters) from this house ever been diagnosed with any of the following conditions? | - 1. ☐ Asthma   2. ☐ Chronic obstructive respiratory diseases (COPD)   3. ☐ Leukaemia   4. None of the above |
| 1. Have you been diagnosed with lung cancer? | 1. Yes 2. No |
| 1. Have anyone from your house been diagnosed with lung cancer? | 1. Yes 2. No |
| 1. Have you lost any family member from this house because of lung cancer? | 1. Yes 2. ☐ No |
| 1. Do you often experience?   *(Tick all appropriate box)* | 1. ☐ Persistent cough 2. ☐ Shortness of breath 3. ☐ Coughing up blood 4. ☐ Unexplained weight loss 5. ☐ Repeated respiratory infections 6. ☐ None of the above |
| 1. If you are invited for a lung cancer screening, will you accept an invitation? | 1. Yes 2. No |
| **TO BE COMPLETED BY THE RESEACHER/ RESEARCH ASSISTANT** | |
| 1. In which room was the detector placed | 1. Living room 2. Bedroom 3. ☐ Other (Specify)___________ |
| 1. Placing radon detectors | Date placed__________Time_______  Date removed________Time_______ |
| 1. Radon detector’s reading | Radon concentration________Bq/m^3^  Temperature______^o^C  Humidity__________  Atmospheric Pressure__________ |
